# Piloting the use of objective structured clinical examinations (OSCEs) to assess undergraduate medical students’ clinical competence in psychiatry in Zimbabwe

**DOI:** 10.1192/bji.2021.49

**Published:** 2022-08

**Authors:** Tatenda Ruwizhu, Ropafadzo Nyamukapa, Fungisai Mazhandu, Julia Mutambara, Walter Mangezi, Susannah Whitwell

**Affiliations:** 1 MMed, Consultant Psychiatrist, Department of Psychiatry, Midlands State University, Zimbabwe. Email: tatendaruwizhu@gmail.com; 2 MBChB, Psychiatry Registrar, Department of Psychiatry, Midlands State University, Zimbabwe; 3 MMed, Consultant Psychiatrist, Department of Psychiatry, Midlands State University, Zimbabwe; 4 PhD, Senior Lecturer, Department of Psychiatry, Midlands State University, Zimbabwe; 5 MMed, Consultant Psychiatrist, Department of Psychiatry & Department of Health Professions Education, University of Zimbabwe, Zimbabwe; 6 MBBS, MRCPsych, MSc, Consultant Psychiatrist, South London and Maudsley NHS Foundation Trust, London, UK

**Keywords:** Low- and middle-income countries, curriculum development, collaboration, undergraduate education and training, medical students

## Abstract

This report describes a pilot project which involved undergraduate medical students’ clinical competence in psychiatry assessed through objective structured clinical examinations for the first time in Zimbabwe. The pilot describes how gaps in medical education can be addressed by collaborative partnerships that allow sharing of knowledge by local institutions and international experts.

Clinical competence in psychiatry can be assessed using various methods. These include long cases, short cases and objective structured clinical examinations (OSCEs). OSCEs are assessments of clinical skills in a simulated environment and have the advantage of testing multiple clinical skills using a standardised rating of performance by multiple raters. They are useful for examining areas where suitable patients are not available at the time of the examinations. Students complete a circuit of ‘stations’, each with a standardised simulated clinical scenario using an actor who simulates a patient and rated by an examiner using a standard rating scale.^1,2^ Long cases as assessments have been replaced in many countries owing to concern about the unreliable nature of the examination.

Long cases are the current clinical assessment for medical students undergoing psychiatry placements in Zimbabwe. Following a 6-week psychiatry clerkship, students are required to sit a clinical examination consisting of an unobserved 1 h clerking of a psychiatric patient followed by a presentation of their findings to a panel of at least two examiners and answering unstructured questions about the case. Marks are allocated for history taking, mental state and physical examinations, diagnostic formulation and case discussion. Disadvantages of long cases include inter-case variance, inter-examiner variance and unstandardised rating of student performance, with a single case/clinical problem that is assessed rather than range of clinical skills.^3^

OSCEs are used in other undergraduate placements at medical schools in Zimbabwe but not in psychiatry. Reasons cited by faculty members for not using OSCEs in psychiatry included psychiatry examiners’ lack of experience in conducting OSCEs and a high student:staff ratio that poses logistical challenges in setting up OSCEs with multiple stations. Concerns about budgetary restrictions in the training and payment of actors used as simulated patients were also raised. Despite similar challenges, training institutions in other low- and middle-income countries (LMICs) have collaborated with medical educators from high-income countries in teaching and examining medical students^4,5^ and there was a need to explore the feasibility of this in introducing OSCEs as psychiatric placement assessments for medical students in Zimbabwe.

In 2010 there was a national undergraduate curriculum review as Zimbabwe was moving from having a single medical school towards setting up a medical school in each of the ten provinces. This raised the issue of standardising the teaching and assessment of students. Zimbabwe received a Medical Education Partnership Initiative (MEPI) capacity-building grant in partnership with the University of Colorado at Denver, Stanford University, the University of Cape Town, University College London and King's College London. Through the collaboration, the Novel Education Clinical Trainees and Researchers (NECTAR) programme was designed which spearheaded the undergraduate curriculum review and a move towards a competency-based curriculum.^6^ Trainees from this programme led the pilot of psychiatry OSCEs at the Midlands State University (MSU), which is one of the three institutions training medical students in Zimbabwe. Psychiatry training is conducted in the fourth year of the 5-year undergraduate medical programme with clinical attachments in the Mental Health Department at Gweru Provincial Hospital. The OSCE pilot was part of curriculum development and contributed to low-stakes formative assessment to eventually become a high-stakes summative assessment. The OSCE pilot included several stages that are outlined below.

## The stages of the pilot

### Training of examiners

Faculty members and an external examiner from the University of Zimbabwe were trained to conduct OSCEs by an expert in medical education from the United Kingdom who had experience in setting up and conducting OSCEs in psychiatry in another LMIC. Training was through a workshop and reading material on the background of OSCEs, standards setting, use of scoring forms and the professional conduct of examiners. An OSCE lead responsible for coordinating the examinations was selected.

### Preparation of actors/simulated patients

The simulated patients were psychiatric nurses and intern psychologists who had an understanding of different psychiatric conditions based on their working experience. They were trained through a workshop conducted 1 week before the examinations. Besides role-play, the simulated patients also received training on confidentiality and professional conduct of examinations. Specific scripts of the roles for the examination were given 3 days before the examination and the simulated patients had practice sessions with the OSCE lead a day before the examinations.

### Preparation of students

There was a briefing for students on the OSCE as part of their assessment at the start of their psychiatry placement. Students were given opportunities to practise their clinical skills while being observed by peers and tutors in ward rounds and they also practised through role-play. A week before the examinations, the students had a tutorial on the processes they would follow in the OSCE, including rating of performance through sharing of the marking scheme.

### Setting up the OSCE circuit

A total of 17 students were examined in two runs. The first was conducted in June 2019 with eight students and the second was in November 2019, when nine students were examined. The OSCEs were conducted in an out-patient clinic that provided several rooms in close proximity to allow students to circulate easily.

As a feasibility project, five OSCE stations were set up and each had 10 min allocated. Skills tested were history taking, conducting a mental state examination, risk assessment and giving information to patients. The range of conditions used included psychosis, dementia, depression, alcohol misuse and HIV-related mental disorders. The students were given 2 min to read instructions on the door of the examination room before entering and they also had a copy of the instructions for reference on the desk used in the room.

### Consent

This pilot was part of curriculum development and the students, simulated patients and examiners gave consent for their feedback to be published.

## Results

### Students’ experience

Feedback on the students’ experience of the OSCEs was obtained through a group discussion with the students a day after the examinations, and more than two-thirds (12) of the students participated. The students said that it was their first experience of having their interview skills examined, as OSCEs in other disciplines involved physical examination of patients and interpretation of results of investigations. They felt that the OSCEs were well organised and timekeeping was well done. However, some students said that some stations had too much time to perform the tasks that were given and others reported that they found some instructions to be unclear and it was difficult to know exactly what to do.

The students said that they had expected the examiners to ask questions to clarify their findings as they did in the long cases and they were unsure how to proceed without any questions asked. They were impressed with the performance of the actors as they sounded like real patients.

### Examiners’ experience

The examiners gave feedback in the examiners meeting following the OSCEs. They reported that they had an overall good experience as they used standardised scores to allocate students, which was different from the long case assessments.

They felt that the students showed poor follow through in their questioning of the patients and some questions sounded memorised without considering the patients’ responses. The examiners noted that some students with good clinical acumen had a poor bedside manner and this was seen in the OSCEs but could not be assessed through the long case examinations. They noted that some students kept pausing and looking at the examiners, expecting feedback during the interview with simulated patients. Examiners expressed that some stations did not allow adequate discrimination between different students' performances and needed a wider range of marks.

Overall, examiners reported that the simulated patients were excellent in their roles. Examiners filled in actor assessment forms that asked about their consistency of answers, depiction of roles and overall performance and all the actors scored ‘good’ to ‘very good’ in the different categories. Examiners expressed the need to give the actors a break, possibly after every five students, to prevent exhaustion.

Examiners reported that the pilot showed how collaborative psychiatry teaching and assessment of medical students in training institutions in Zimbabwe could be conducted. They also indicated that support from colleagues with experience in conducting OSCEs was invaluable and the partnerships should continue as they had a positive impact on medical education in psychiatry in Zimbabwe.

### Simulated patients’ experience

Simulated patients gave feedback a day after the examinations in a group discussion with the examiners. They said that they pictured patients with similar conditions who they had treated and they expressed themselves as those patients. They reported that they could tell which students were doing well and which ones seemed to be struggling in asking the right questions and expressing themselves. They also noted that although some students were asking relevant questions, they had a poor bedside manner which could be improved.

## Lessons learned

The piloting of OSCEs showed that it is possible to have standardised assessments in psychiatry for undergraduate medical students in Zimbabwe. Although the pilot was at an institution with a small number of students compared with other universities, OSCEs can also be conducted for large numbers of students and several runs can be organised, with continued exploration of innovative ways of performing standardised examinations in low-resource settings.

The pilot was the first time that simulated patients were used in MSU psychiatry examinations and they enabled standardised assessments of student performance. Although more challenging to set up than long case examinations, as they are costly and involve training patients and examiners, OSCEs provide standardised assessments in a safe and controlled environment. The simulated patients were drawn from a pool of healthcare professionals who can also be trained to objectively assess the students. Healthcare workers and other students in different health disciplines can be trained to perform the roles of simulated patients in low-resource settings where the use of actors may be more expensive. Simulated patients can also be included in the long case examination if complete OSCE circuits cannot be conducted. The use of simulated patients has the disadvantage of creating textbook-like patients, which students are unlikely to encounter in clinical settings. However, using real patients in psychiatry OSCEs poses ethical challenges, including obtaining consent with mentally unstable patients and eliciting standardised responses. If stable patients are used (e.g. from out-patient clinics), asking them to repeatedly simulate scenarios could be distressing even if they may portray their own experience. In this pilot, unlike professional actors, the healthcare workers who simulated the patients had experience of interaction with real patients and this helped in depicting them. With training, simulated patients could also rate students’ performance and this could lead to the introduction of stations that do not need an examiner. Exploration of the correlation of student performance ratings between simulated patients, faculty examiners and possibly other health workers in psychiatry settings in LMICs could lead to combined assessors and help address the limited number of faculty examiners. Other health workers could be more involved in the rating of students in countries like Zimbabwe, where challenges faced by psychiatry departments include the high student:staff ratio. Clinical psychologists, occupational therapists and psychiatric nurse practitioners are already part of the faculty and involved in undergraduate psychiatry teaching and assessments. There is a need for student, faculty and simulated patient feedback forms with rating scales to get comparative experiences in future examinations and these can be used for quality improvement.

Collaboration between medical educators from institutions in high-income countries and LMICs can offer an opportunity to address gaps in knowledge and skills to provide evidence-based assessments in psychiatry teaching.

To the best of our knowledge there is a paucity of literature on whether OSCEs in psychiatry are being conducted in LMICs and information on how different faculties have implemented them. Our report adds to the body of literature practical experiences on how we have implemented the different strategies highlighted in the literature and we hope that it can help other medical schools in LMICs in implementing and improving reliable assessments in psychiatry education.

## Data Availability

The data that supports the findings of this report is available on request from the corresponding author. It is not publicly available owing to the potential compromise of the privacy of student assessment scores.
